# Human hand kinematic data during feeding and cooking tasks

**DOI:** 10.1038/s41597-019-0175-6

**Published:** 2019-09-05

**Authors:** Alba Roda-Sales, Margarita Vergara, Joaquín L. Sancho-Bru, Verónica Gracia-Ibáñez, Néstor J. Jarque-Bou

**Affiliations:** Department of Mechanical Engineering and Construction, Universitat Jaume I., 12071 Castelló de la Plana, Spain

**Keywords:** Orthopaedics, Translational research, Biomedical engineering

## Abstract

This work presents a database of human hand kinematics containing data collected during the performance of a wide variety of activities of daily living involving feeding and cooking. The data were recorded using CyberGlove instrumented gloves on both hands measuring 18 degrees of freedom on each. A total of 20 subjects participated in each part of the experiment, and the objects and their arrangement were the same across subjects, although they performed the tasks in a natural non-directed way. This dataset contains a total of 1160 continuous calibrated recordings taken at 100 Hz during the performance of the tasks, with filtered signal. Statistical descriptive analyses from these data are presented. This database can be useful for machine learning purposes and prostheses control, as well as for the characterization of healthy human hand kinematics.

## Abstract (summary)

**Table:** 

Design Type(s)	modeling and simulation objective • observation design
Measurement Type(s)	hand kinematics
Technology Type(s)	sensor
Factor Type(s)	biological sex • handedness
Sample Characteristic(s)	Homo sapiens • manus

Machine-accessible metadata file describing the reported data (ISA-Tab format)

## Background & Summary

The hand is a complex system, with many degrees of freedom (DoF), that enables humans to perform a large variety of grasping and manipulation actions required in activities of daily living (ADL), using a wide range of objects. Hand kinematics is being studied for purposes such as characterizing healthy hand movement patterns^1^, assessing patients’ abilities^2^ or the effect of object design on grasping^3^. Furthermore, with the rise in robotics and prosthetics, it has become crucial for the development of anthropomorphic systems^4^. For these purposes, and because of the versatility of the hand, a large amount of kinematic data (for all hand DoF) is needed to cover the interaction with the different objects used in different environments. Continuous recording of kinematics is essential to characterize the range of motion and velocities required for the different phases of reaching, grasping, manipulating and releasing. Moreover, data presented as anatomical angles are more meaningful and facilitate the comparison of data from different experiments independently of the motion capture system used. In this sense, several researchers^5^ have pointed out the importance of high-quality open-access datasets of grasping data, while also highlighting the need to compile, classify and standardize these data.

The *Hand Corpus* open repository (http://www.handcorpus.org) was created to undertake these goals, as it allows scientists to share grasping and manipulating data collected using different motion capture technologies. Nevertheless, the datasets in this repository, as well as the other datasets in the literature, present some weaknesses regarding their usability in machine learning, hand kinematics characterization or clinical evaluation. Some datasets offer limitations regarding the amount of data presented, are limited to grasp type classification^6,7^ or consider hand kinematics from just three markers on the hand^8^. Furthermore, datasets with several DoF present other limitations:

- **Tasks**: Only reaching and grasping movements^9–14^, static grasp postures^9,10,12,14–17^ or exploratory/haptic tasks^18^ were recorded during product manipulation. These tasks lack representativeness of ADL because of the limited range of activities considered but also because subjects performed the tasks following precise instructions.
- **Objects used**: Some of the datasets recorded tasks simulating the use of objects, but not using any object^11,15–17,19,20^.
- **Type of data presented**: Some datasets only provide raw data from the motion capture system (cameras or gloves)^12,19,20^ instead of offering anatomical angles.
- **Number of subjects**: Some of those datasets provide data from only one subject^9,11,15–17,19,20^.
- **Number of hands studied**: All the datasets cited only studied subjects’ dominant hand.

Table 1 shows an overview of different datasets focused on hand kinematics and their characteristics.

**Table 1:** Main characteristics of datasets focused on hand kinematics.

Dataset	Objects	Subjects	Tasks	Motion capture system	Type of data
NTUA^9^	4	1	Static grasps, reach and grasp	CyberGlove	Joint angles (20 DoF)
UNIPI^15,29^	Imagined	1	Static grasps	Phase Space	Joint angles (15 DoF)
UNIPI-ASU^1,16^	Imagined	1	Static grasps	CyberGlove	Joint angles (15 DoF)
DLR^1,10^	23	7	Static grasps, reach and grasp	Vicon	Joint angles (20 DoF)
DLR^17,30^	None	1	Static postures	MRI	Hand model (24 DoF)
UNIPI^11,31^	Imagined	1	Reach and grasp	Phase Space	Joint angles (24 DoF)
UNIPI^19,31^	None	1	Free space	Phase Space	Raw data (24 DoF)
UNIPI^20,31^	None	1	Free space	Phase Space	Raw data (26 DoF)
TU Berlin 1 – IJRR^12,32^	14	5	Static grasps, reach and grasp	CyberGlove	Raw data (23 DoF)
UNIPI^18,31^	2	1	Haptic exploration	Phase Space	Joint angles (26 DoF)
HUST^33,34^	14	30	Reach and grasp	CyberGlove	Joint angles (16 DoF)
TUB^35,36^	25	17	Reach and grasp	CyberGlove	Raw data (21 DoF)
UNIPI^13,37^	21	6	Reach and grasp	Phase Space	Joint angles (20 DoF)
NINAPRO^14,38^	16	78	Static grasps/postures	CyberGlove	Raw data (22 DoF)

In this paper we present the KINE-ADL BE-UJI Dataset^21^, which contains a total of 1160 recordings with anatomical angles of both hands while performing feeding and cooking activities using a large variety of products. Experiments were performed by 20 healthy subjects while wearing CyberGlove instrumented gloves on both hands, 18 DOF being recorded in each hand at a frequency of 100 Hz. The main contribution of this dataset compared to others is the variety of objects used (66 objects), the in-depth study of representative feeding and cooking tasks (58 tasks, divided into 178 actions) and the freedom given to the subjects to perform the tasks. Moreover, the data were collected from both hands, which allows the study of hand coordination. It is also important that the sample of subjects was selected so as to be representative of the healthy adult population (with a controlled proportion of laterality and gender). Furthermore, the data presented is standardized, as it is presented as anatomical angles following the ISB sign criteria^22^. The dataset consists of a Matlab/GNU Octave data structure (.*mat*) (*provided also in* .*csv format*) with kinematic data and data about the subjects recruited (age, gender, laterality, weight, height, hand length, hand width and active range of motion (AROM) measured for each DoF). This .*mat* file is accompanied by a guide where information regarding the environment, tasks, objects, data acquisition system and file structure is detailed, thereby allowing the classification of information regarding these parameters.

## Methods

### Study participants

The study consisted of two experiments (A and B), with 20 subjects (10 males, 10 females) participating in each experiment. Only 15 subjects participated in both experiments, so that the total amount of subjects recruited was 25. In both experiments, two of the 20 subjects were left-handed. The mean age of subjects recruited was 35.5 ± 7.67 years in experiment A and 38.05 ± 9.52 years in experiment B. The criteria used to select subjects were gender parity in overall data, age between 20 and 65, no reported upper limb pathologies and laterality representative of the overall population (20% of data from left-handed individuals). Before the experiments, all participants gave their written informed consent. All the experiments were performed in accordance with the Ethics Committee of the Universitat Jaume I.

### Acquisition setup

#### Instrumentation

Data acquisition was performed using two CyberGlove (CyberGlove Systems LLC) instrumented gloves (CyberGlove II on the right hand and CyberGlove III on the left hand) connected to a laptop. Each of these gloves has 18 strain gauges that allow the anatomical angles of the underlying joints to be determined. The angle rotated by each joint with respect to the reference posture (hands resting flat on a table, with the fingers and thumb close together, and the middle fingers aligned with the forearms) is then calculated from these signals, according to a previously validated calibration protocol^23^. Furthermore, all the experiments were recorded on video, so as to be able to check the performance of the task when subsequently required.

#### Environment

**Fig. 1:**
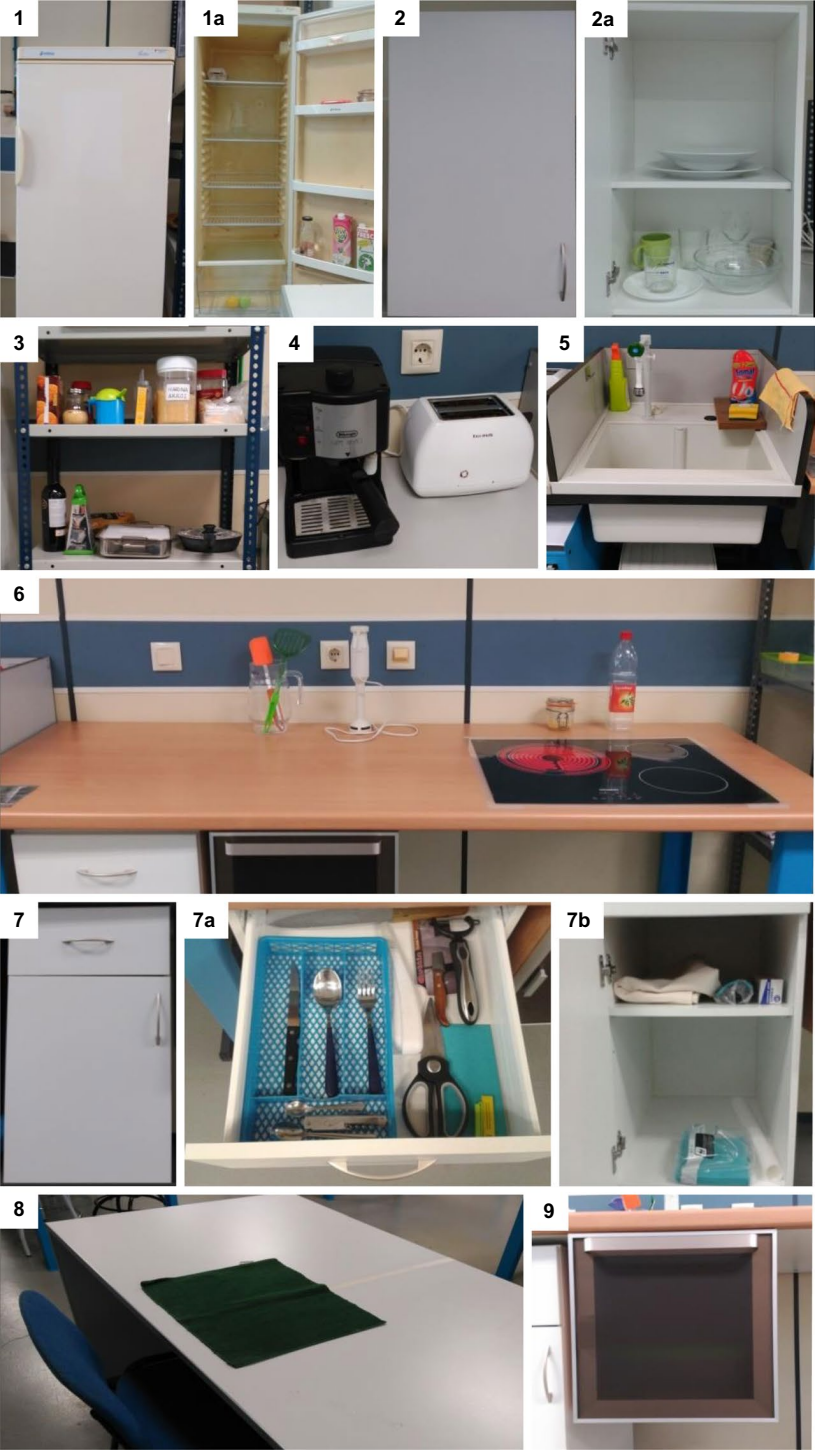
Different scenarios of the experiment. Scenarios: Refrigerator (1), high cabinet (2), shelves (3), small worktop (4), sink and a rubbish bin (5), large worktop (6), low cabinet with a drawer in its upper part and shelves in the lower part (7), a table and a chair (8), and an oven (9).

The tasks were performed in a laboratory, within an environment that simulated a kitchen (Fig. 1), composed of: a refrigerator (Scenario 1), a high cabinet (Scenario 2), shelves (Scenario 3), a small worktop (Scenario 4), a sink and a rubbish bin (Scenario 5), a large worktop (Scenario 6), a low cabinet with a drawer in its upper part and shelves in the lower part, which has a door (Scenario 7), a table and a chair (Scenario 8) and an oven (Scenario 9).

#### Objects

**Fig. 2:**
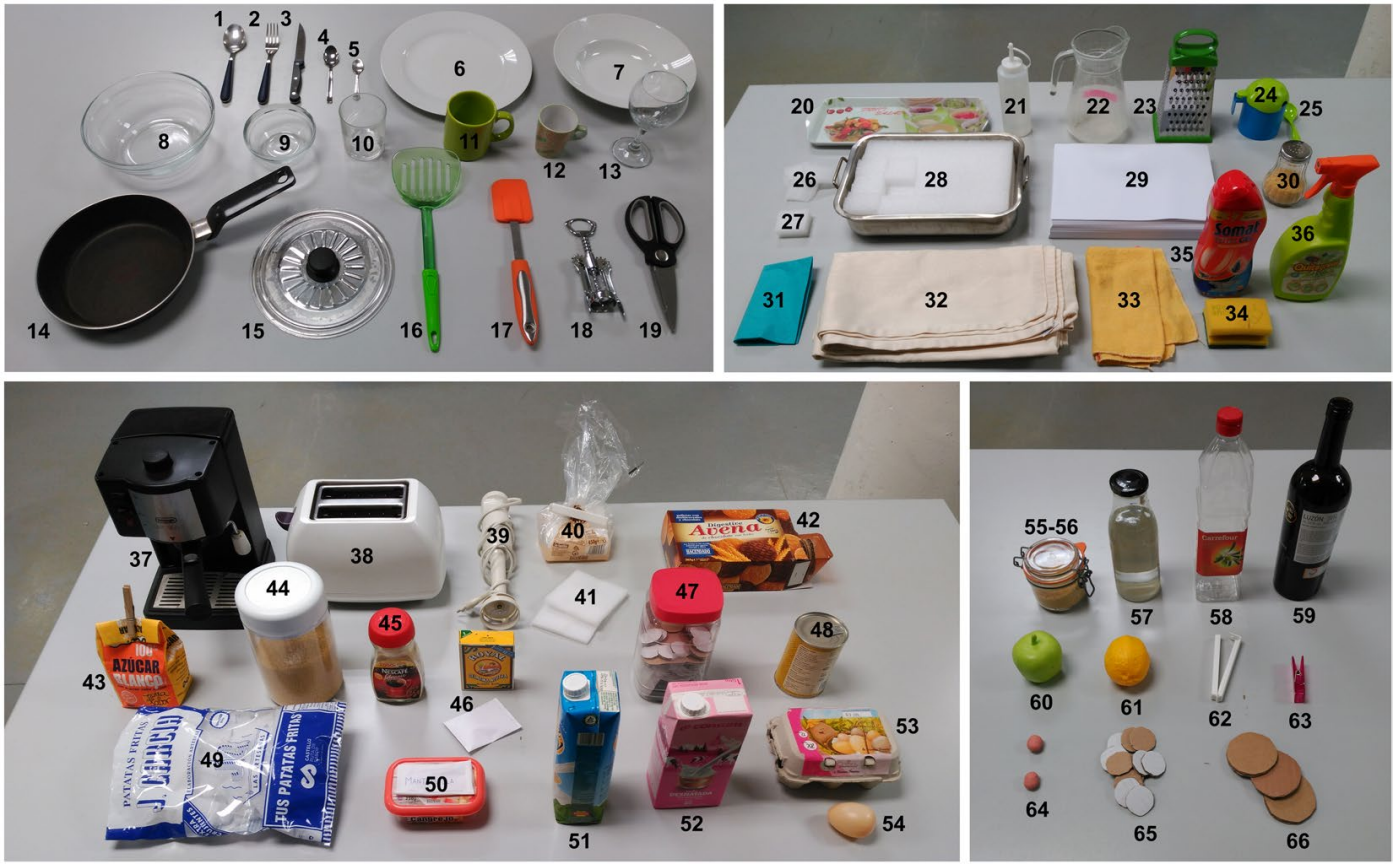
Overview of the objects used during the experiments. Objects labelled as in the guide attached to the dataset.

A total of 66 objects were used to perform the tasks in the experiments (further information regarding their characteristics can be found in the guide attached to the dataset). The objects were chosen so as to be representative of those most commonly used in cooking and feeding tasks, and were checked to ensure they covered the cooking and feeding objects from the *Yale-CMU-Berkeley Object and Model Set*^24^, proposed by Calli *et al*. Some of the objects used were not real, in order to prevent the gloves from getting stained or wet. For example, the eggs to be broken had been previously emptied through a small hole made in the shell. All liquids were replaced by water, and materials such as flour or sugar that could have stained the gloves were replaced by durum wheat semolina. Pieces of polystyrene or cardboard were used to simulate biscuits, bread or crisps. The initial location of the objects in each scenario can be found in the detailed guide attached to the database. Figure 2 shows an overview of the objects used.

### Acquisition protocol

The main dimensions of the hands were measured before helping the subject to put on the instrumented gloves following the manufacturer’s instructions. Participants were given clear instructions about how to perform the task, and they were told to start and end each task in the same posture: hands lying relaxed at both sides of the body for tasks performed in a standing posture, and hands lying relaxed on the table when sitting. While carrying out each task, the operator marked (or labelled) the time stamp of some specific events (using the glove software) that were later used to separate different phases or actions.

#### Recorded tasks

Two experiments (A and B) were performed. In experiment A, the activities performed were: preparing and having breakfast, baking a cake and cooking omelets. In experiment B, the activities were: setting the table, clearing the table and washing the dishes, making coffee and preparing a simple meal, considering the whole process of performing each task (taking the products from the different scenarios, transporting them, opening/using them and, in some cases, putting them back in their place). Furthermore, all these tasks were separated into different recordings (e.g. using the toaster or pouring and drinking milk), and these recordings were also separated into different elementary tasks (e.g. object grasping, manipulation such as opening tins/jars/bottles, transportation of objects, pouring liquid/solid substances, eating/drinking and other relevant actions). Therefore, experiment A was divided into 33 recordings and experiment B consisted of 25 (a description of all the recordings can be seen in Tables 2 and 3). Further information regarding the elementary tasks considered in each recording can be found in the guide attached to the dataset.

**Table 2:** Recordings in experiment A, where R is the ID number of the recording (100 onwards belong to experiment A), and S indicates whether the activity was performed sitting (x) or not.

R	S	Preparing And Having Breakfast
101		Using a toaster.
102		Setting the table: placing the toast.
103		Setting the table: placing a box of biscuits, a carton of milk and an apple.
104		Setting the table: placing a jar of jam, a tub of butter, a mug and a glass.
105		Setting the table: placing a spoon and a knife and sitting on the chair.
106	x	Pouring and drinking milk.
107	x	Dipping a biscuit in milk and eating it.
108	x	Pouring and drinking juice.
109	x	Spreading butter on toast.
110	x	Spreading jam on toast and eating it.
111	x	Eating (simulated) the apple.
		**Preparing**, **Baking and Eating A Cake**
112		Carrying utensils and ingredients to the worktop: a bowl, a carton of eggs and a lemon.
113		Carrying ingredients to the worktop: a jar with flour in it, a bag of sugar and a box of baking powder.
114		Carrying utensils and ingredients to the worktop: a carton of milk and a glass.
115		Breaking an egg into a bowl and throwing the eggshell into the bin.
116		Beating the egg with a fork.
117		Filling a glass with sugar.
118		Grating a lemon.
119		Filling a glass with flour.
120		Opening a carton of milk with scissors and pouring milk.
121		Pouring baking powder into the bowl.
122		Using a mixer to mix the ingredients for the cake dough.
123		Pouring the cake dough onto the baking tray and using a spatula.
124		Putting the baking tray into the oven. Taking the baking tray out of the oven.
125		Cutting a piece of cake with a knife and eating it (simulated).
126		Putting the spatula, the knife, the bowl, the glass and the grater in the sink.
127		Carrying the carton of eggs, the lemon and the carton of milk back to the fridge.
128		Carrying the jar of flour, the bag of sugar and the baking powder to the shelves.
129		Putting the tray with 3 kg of food on it into the oven. Taking the tray out of the oven.
		**Preparing Omelets**
130		Beating an egg and salting it.
131		Preparing the pan for cooking on the hob.
132		Cooking and serving a small omelet.
133		Cooking, serving and cutting a big omelet.

**Table 3:** Recordings in experiment B, where R (200 onwards belong to experiment B) is the ID number of the recording and S indicates whether the activity was performed sitting (x) or not.

R	S	Setting The Table
201		Putting a tablecloth on the table.
202		Placing a dish, a glass, a fork, a knife and a napkin.
203		Placing a jug of water, an oil cruet, a salt-shaker and a bowl.
		**Clearing the Table and Washing the Dishes**
204		Putting the glass, the jug, the oil cruet and the salt-shaker back in their place.
205		Throwing the leftovers on the plates into the rubbish bin.
206		Throwing the leftovers in the bowls into the rubbish bin.
207		Removing the tablecloth from the table and folding it.
208		Washing the glass, the bowl, the dish, the fork and the knife.
209		Putting the glass, the bowl, the dish, the fork and the knife back in their place.
210		Cleaning the worktop.
		**Preparing and Drinking Coffee**
211		Taking a jar of ground coffee and opening it.
212		Filling the filter handle of the coffee machine with coffee.
213		Placing a cup under the coffee machine and pressing the power button.
214		Placing the cup of coffee and the sugar pot on a tray. Carrying it to the table.
215		Throwing the used ground coffee into the rubbish bin.
216	x	Adding sugar to the coffee, stirring and drinking it (simulated).
		**Preparing and Eating a Simple Meal**
217		Pouring crisps from a bag into a bowl.
218		Closing the bag of crisps with a sealing clip.
219		Pouring olives from a tin into a little bowl.
220		Pouring salted biscuits from a jar onto a dish.
221		Setting the table: placing the dish and the bowls.
222		Opening a bottle of wine with a corkscrew.
223		Setting the table: placing a glass of wine. Sitting on the chair.
224	x	Pouring wine and drinking it (simulated).
225	x	Eating (simulated) olives, crisps and biscuits.

Some of the recordings were performed with the subject standing and others while sitting on a chair (as specified in Tables 2 and 3). Only the eating or drinking activities were simulated, by just bringing the food close to the mouth, and this has been indicated in the task description. The rest of the tasks were performed with realistic objects, and subjects were free to perform the tasks in the way they preferred.

#### Elementary tasks

As mentioned previously, each of the recordings (R) (33 recordings in experiment A and 25 in B) is composed of different elementary tasks. For example, in the activity of having breakfast (consisting of 11 records, as seen in Table 2) record R = 106 (pouring and drinking milk) is composed of four elementary tasks: opening the carton, pouring, closing the carton and drinking (see Table 4). For an unambiguous identification of each of the tasks, a unique ID was assigned to each elementary task, with a total of 99 elementary tasks in experiment A and 79 in B (178 elementary tasks altogether). All the elementary tasks involved grasping or manipulating a product or element with the hands, except for some cases where the subject moved without handling anything, which were labelled as “Displacement without manipulation”. For each elementary task, the record considers all time instants since the object was grasped until it was released. In those cases in which the object was released in a specific place or transported to a specific location in the scenario, this location is specified in the description of the elementary task. In all other cases, the release was performed on the surface closest to the subject (table, worktop, etc.).

**Table 4:** Elementary tasks into which task R = 106 is divided. Columns containing R (ID of the recording), ID (ID of the task), OBJ (ID of the objects used during the task), SCEN (ID of the scenario where the task is performed) and S (marked with an “x” when the task was performed sitting).

R	ID	OBJ	SCEN	S	Having Breakfast
106	16	51	8	x	Opening the cap of the carton of milk
	17	11, 51	8	x	Pouring milk from the carton into the mug
	18	51	8	x	Closing the carton of milk
	19	11	8	x	Drinking from the mug (simulated)

#### Active range of motion (AROM)

After performing all the experiments, subjects were asked to perform a set of postures^25^ in order to measure their AROM of the joints of both hands, which are presented in the .*mat* file, where subject information is also provided.

### Signal processing

#### Angles calculation

**Fig. 3:**
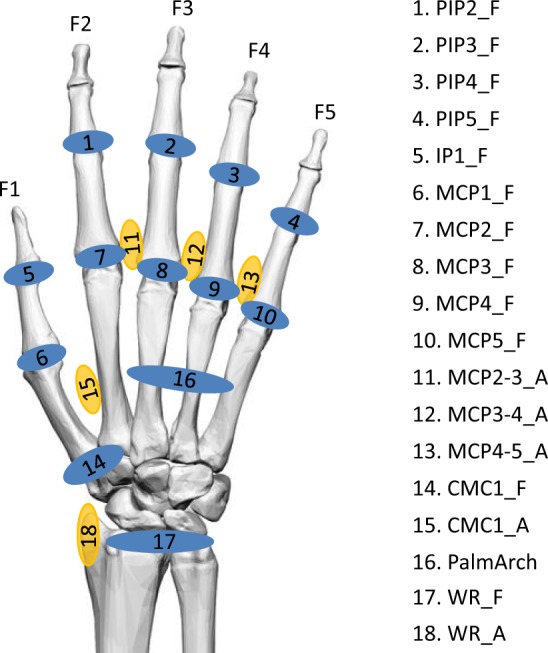
List of recorded anatomical angles. Nomenclature: _F for flexion (in blue), _A for abduction (in yellow); 1 to 5, digits. Joints: IP for interphalangeal joint, PIP for proximal interphalangeal joints, MCP for metacarpophalangeal joints, CMC for carpometacarpal joints, PalmArch for palmar arch resulting from flexion/extension of carpometacarpal joints of ring and little fingers, WR for wrist.

Joint angles were calculated from raw data collected according to the calibration protocol proposed in previous works^23^. This protocol includes the determination of gains and also some corrections because of cross-coupling effects for specific anatomical angles. The anatomical angles obtained according to the protocol are those shown in Fig. 3.

#### Data cutting and splitting

The initial and final instants of each record, in which the hands were static, were trimmed. The records were then separated into the different elementary tasks as detailed in the dataset guide by using the labelling performed by the operator while recording the data. In some specific cases in which labelling data was missing, labelling was performed using the video recordings.

#### Filtering

All data were filtered with a 2^nd^ order two-way low pass Butterworth filter with a cut-off frequency of 5 Hz.

## Data Records

### Volume of data collected

A total of 3560 elementary tasks were recorded across all the subjects and experiments, with a total duration of the recordings of 7 h, 30 min and 43 seconds.

### Data files

Data is presented as a single Matlab data structure (BE_UJI_DATASET.*mat*)^21^, which is composed of two secondary structures (KINEMATIC_DATA and SUBJECT_DATA). KINEMATIC_DATA contains all kinematic data recorded, classified by experiment, record, part and subject, while SUBJECT_DATA contains data of the subjects recruited (age, gender, laterality, weight, height, hand length, hand width and measured AROM). This structure is accompanied by a guide (.pdf), which provides detailed information regarding the data series as well as the environment, tasks, objects and data acquisition system.

### Sign criteria

The sign criteria used on each joint movement were defined as follows:

**PIP**(**2–5**)**_F**, **IP1_F**, **CMC1_F**, **MCP**(**1–5**)**_F**, **WR_F**: Flexion+/Extension−

**MCP**(**2-3**, **3-4**, **4-5**)**_A**: Fingers separated+/Fingers together−

**PalmArch**: Flexion+/Extension−

**WR_A**: Ulnar deviation+/Radial deviation−

**Fig. 4:**
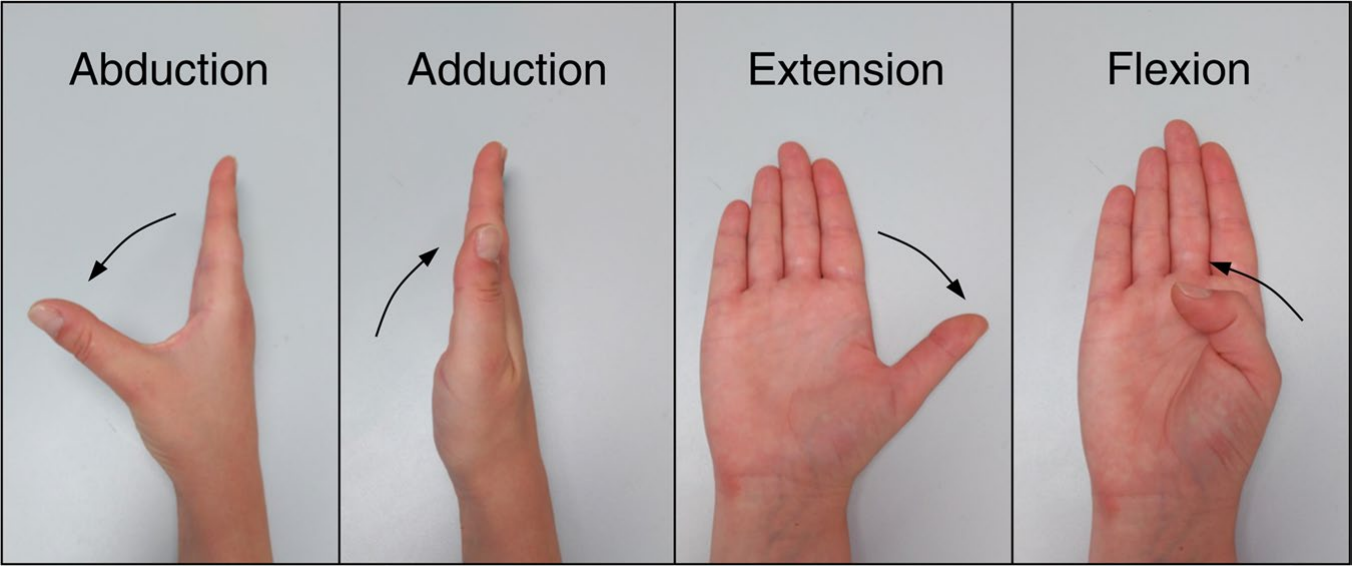
Movements of the carpometacarpal joint.

**CMC1_F**: Flexion+/Extension− (See Fig. 4)

**CMC1_A**: Abduction+/Adduction− (See Fig. 4)

Notice that movement of thumb CMC joint is complex, and nomenclature used in literature to define these movements is varied^26,27^. We adopted the one used by Brand and Hollister^27^.

## Technical Validation

### Data acquisition

Before and after carrying out each experiment the subjects were asked to perform movements such as closing their hands or just moving them randomly, in order to make sure that all the gauges were shown to be working on the virtual model of the CyberGlove software.

Furthermore, all tasks recorded were checked in order to ensure that the number of labels used to divide them into elementary tasks was correct and that no labels were missing.

In order to avoid possible unexpected signal values, all data collected were filtered using a 2^nd^ order two-way low pass Butterworth filter with a cut-off frequency of 5 Hz, as explained in previous sections.

### Comparison of active and functional range of motion for each subject and experiment

The percentiles P95 and P5 were calculated for each hand joint, experiment and subject. Then, for each subject and experiment, a subject-specific functional range of motion (FROM) was computed for each hand joint angle as the P5 and P95 percentiles of all his/her recordings, therefore representing the angles of 90% of the postures performed by the subject during the experiment. These FROMs were compared with the AROMs measured for each subject. Almost all the FROMs were inside the AROMs, except in some cases where the extension of thumb interphalangeal and metacarpophalangeal joints and the index metacarpophalangeal joint extension were higher than the AROM measured (maximum difference reported between FROM and AROM was 25° approx.). This may be attributable to activities that implied a passive extension of these joints while manipulating objects (e.g. cutting with a knife implies a precision grasp with a forced extension of the thumb joints and index interphalangeal joint that is higher than the achievable active extension).

### Statistical descriptive analysis of all data collected

**Fig. 5:**
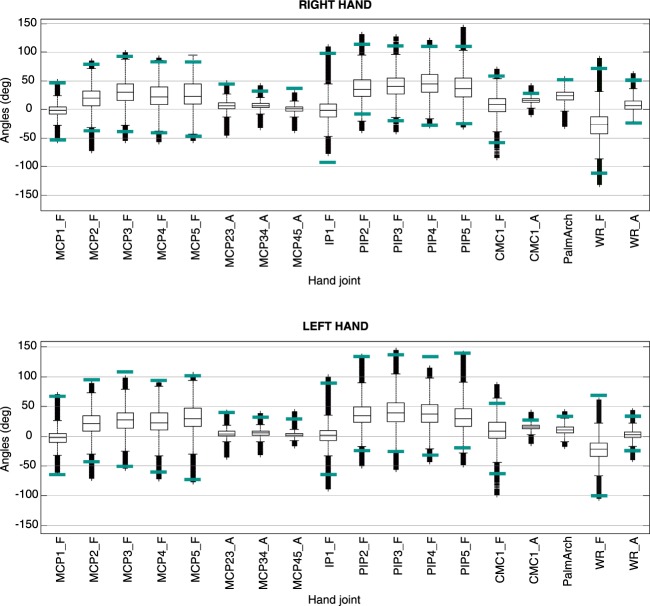
Comparison of maximum AROMs and FROMs. Box and whisker plots for general FROMs, general AROMs are marked with green lines. Unmarked AROMs were not measured. Joints and movements labelled as explained in Fig. 3.

With all data collected, box and whisker graphs were plotted and general FROMs were calculated. Then, the extreme values of all the subjects’ AROMs calculated previously were taken to calculate general AROMs. When general FROMs and AROMs were compared, most values of the FROMs were between those of the AROMs, which supports the veracity of the data. Nevertheless, some outliers were higher than those values (Fig. 5), especially in extension of CMC1, MCP2, PIP2 and PIP3 and flexion of right PIP2 to PIP5. This can also be attributable to activities that implied a passive flexion/extension of joints while manipulating, as mentioned before. It has to be emphasized that the FROMs of PIP2 to PIP5 were higher than the AROMs only for the right hand, which is the dominant hand of most subjects.

### Limitations

The use of instrumented gloves may imply some loss of dexterity during the performance of fine manipulation tasks. Nevertheless, this loss of dexterity may not have a significant effect on the ranges of motion, mean postures or movement synergies.

## Usage Notes

These data can be used for several applications, from machine learning purposes to product design. The main strengths of this dataset for these potential uses are the motion capture characteristics (validity of the motion capture system, anatomical joints measured and frequency of acquisition), the structure of the data presented (.*mat*, which allows easy data handling), the variety of objects used (different shapes and weights) and the wide range of cooking/feeding tasks considered.

It has to be taken into account that real food or drinks were not used to perform the tasks in order to prevent the gloves from getting stained or wet (all products are appropriately tagged with the corresponding substitutive material in the dataset guide file). Therefore, tasks involving these elements were simulated and might be performed in a slightly different way than when performed with real food/drink.

Even though tasks and products of this dataset were selected to be representative of the different cooking and feeding tasks, some specific tasks or objects involving fine motor skills were discarded because of the loss of manipulation dexterity that the use of instrumented gloves implied (e.g. opening the thermally sealed plastic layer of precooked food packaging). Some wrist angles are also missing because of improper fitting of the wrist sensors to some subjects’ hands.

Finally, velocity of performance of the tasks might be slightly affected by the loss of dexterity and touch sensitivity resulting from the use of the instrumented gloves.

## ISA-Tab metadata file

Download metadata file

## Data Availability

The custom Matlab code used to calculate joint angles is freely available on Zenodo^28^.
